# The association between overactive bladder and systemic immunity-inflammation index: a cross-sectional study of NHANES 2005 to 2018

**DOI:** 10.1038/s41598-024-63448-3

**Published:** 2024-05-31

**Authors:** Baian Wei, Ying Zhao, Pinli Lin, Wenqiang Qiu, Shusheng Wang, Chiming Gu, Lili Deng, Tewei Deng, Siyi Li

**Affiliations:** 1 The Second Clinical College of Guangzhou, University of Chinese Medicine, Guangzhou, China; 2 The Second Affiliated Hospital of Guangzhou University of Chinese Medicine (Guangdong Provincial Hospital of Traditional Chinese Medicine), Guangzhou, China

**Keywords:** Overactive bladder, Systemic immune-inflammation index, NHANES, Urology, Bladder, Diseases, Urogenital diseases

## Abstract

Current research indicate that inflammation is linked to the development of overactive bladder (OAB). The aim of this study was to examine the correlation between OAB and the systemic immunity-inflammation index (SII) in the USA. We analyzed data from 31,881 participants in the National Health and Nutrition Examination Survey 2005–2018. SII, calculated as platelet count × neutrophil count/lymphocyte count, was categorized into quartiles. OAB was defined by the presence of urge urinary incontinence and nocturia. Weighted logistic regression models were used to examine the independent relationship between SII and OAB, adjusting for demographic factors, kidney function, and diabetes status. The results showed that each tenfold increase in log-transformed SII was associated with an 18% higher odds of OAB (OR 1.18, 95% CI 1.08–1.28) in the fully adjusted model. Compared to the lowest SII quartile, the highest quartile had a 28% increased OAB risk (OR 1.28, 95% CI 1.12–1.47). The positive association between SII and OAB risk was consistently observed across subgroups stratified by age, sex, race, marital status, education, and poverty level. Our study reveals a positive correlation between SII levels and OAB, indicating that higher SII levels are associated with an increased likelihood of developing OAB.

## Introduction

Overactive bladder (OAB) is a common lower urinary tract storage symptom characterized by urinary urgency. Specifically, patients experience a sudden, uncontrollable urge to urinate, often accompanied by increased urinary frequency and nocturia. In some cases, urge incontinence may also occur, yet without other symptoms resulting from urinary tract infections or vesicourethral pathology^1,2^. In the United States, OAB is a prevalent condition. Research reports indicate that the prevalence of OAB among American males is 16%, while among females, it is 16.9%^3^. The prevalence of OAB rises with age and substantially impacts patients’ quality of life, sleep quality and mental health^3–5^. Collectively, the inadequate relief alongside undesirable side effects of current options, paired with increasing OAB population-level burden with societal aging, underline the major healthcare challenges imposed by this disorder. In the United States, healthcare costs for OAB patients were more than 2.5 times higher than for similar patients without OAB. Furthermore, OAB patients with chronic, age-related comorbidities incurred even higher healthcare costs than non-OAB controls with the same comorbidities^6^.

The systemic immune-inflammation index (SII) serves as a pivotal clinical tool for evaluating local immune response and systemic inflammatory status^7^. Extensive research has demonstrated SII’s potential for predicting prognosis in various diseases, including cancer^8^, coronary artery disease^9^, diabetic kidney disease^10^ and stroke^11^. Emerging evidence suggests that immune-inflammatory responses may contribute to the pathogenesis of OAB^12,13^. The levels of inflammatory proteins such as C-reactive protein, prostaglandins, adipokines, and nerve growth factor (NGF) and brain-derived neurotrophic factor (BDNF) in serum and urine were significantly higher in patients with OAB than in the non-OAB population^14–16^. Inflammation can lead to peripheral afferent nerve hyperexcitability, which causes a series of OAB symptoms such as urinary frequency and urgency^17^, suggesting that inflammation may be a cause of OAB. In addition, Koch et al.^18^ studied the urine proteomics of 20 female OAB patients and found that the proteins in the OAB group were mainly related to the “cellular response to stress” and “regulation of apoptosis” pathways, whereas the proteins in the non-OAB group were mainly related to the “immune system” pathway and the “extracellular matrix organization” pathway. They therefore hypothesized that the development of OAB is related to changes in the immune system. However, the relationship between SII, an important indicator of reactive immune-inflammatory responses, and OAB is unclear.

We therefore used publicly available data from the National Health and Nutrition Examination Survey (NHANES) database to explore the relationship between SII use and OAB among survey participants.

## Materials and methods

### Study population

**Figure 1:**
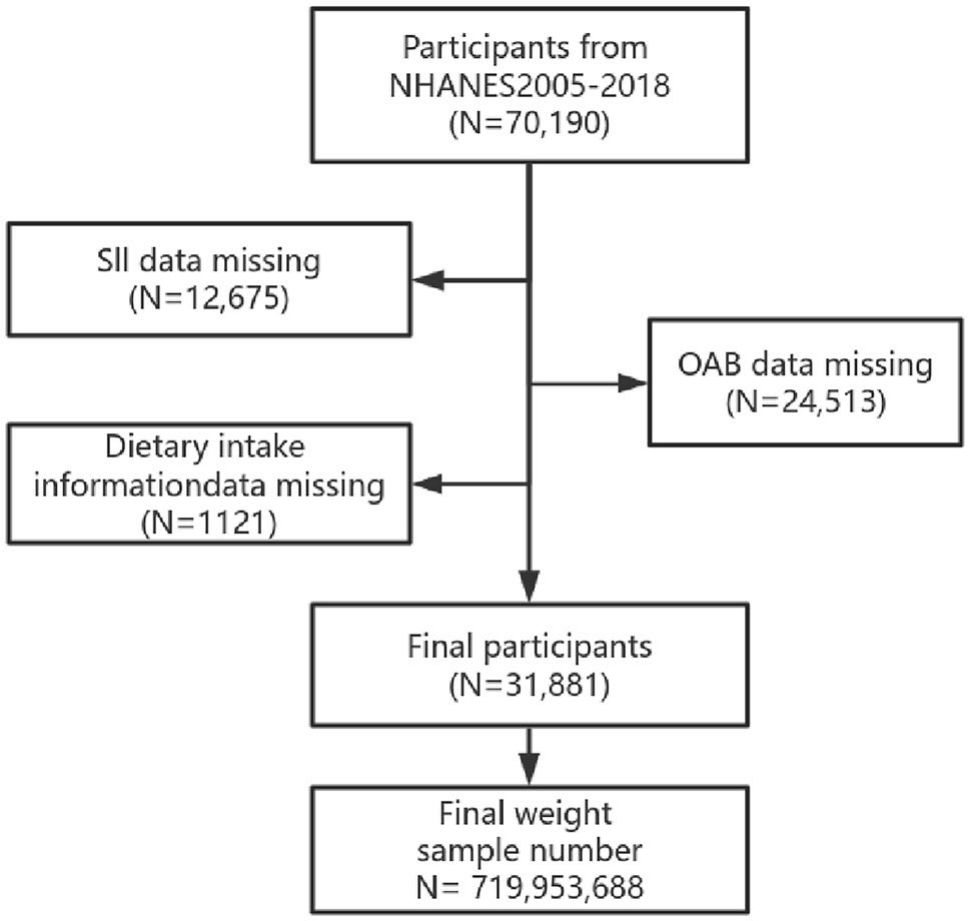
Flowchart of participant selection.

This cross-sectional study included subjects from the nationally representative, continuous Nutrition and Health Examination Survey (NHANES) spanning 2005–2018. Of the 70,190 total participants surveyed over this period, individuals were excluded if they lacked: (1) SII-associated laboratory measurements (n = 12,675), (2) data related to OAB assessment (n = 24,513), or (3) dietary intake information (n = 1121). Ultimately, 31,881 subjects with a combined analytical weight of 719,953,688 were enrolled in the study (Fig. 1).

NHANES applies a complex sampling design involving oversampling to ensure calculated estimates are representative of the U.S. noninstitutionalized civilian population. Sample weights for NHANES 2001–2002 and subsequent 2-year cycles account for the 2000 U.S. Census. Providing all possible weight combinations across multiple cycles would be impractical, so the National Center for Health Statistics supplies guidance on constructing appropriate multi-year weights by simply dividing 2-year weights by the number of aggregated cycles. Adhering to this protocol, we derived weights when combining 2005–2018 NHANES 2-year units to analyze potential associations between OAB and SII over this extended period.

### Variables

#### Demographic characteristics

Sociodemographic characteristics were obtained via home questionnaires. Age was stratified into two groups: 20–59 years, and ≥ 60 years. Race/ethnicity categories consisted of White, Black, Mexican, and Other. Poverty ratio was calculated as monthly family income relative to poverty levels, and dichotomized as: < 1 (low income) and 1–5 or > 5 (middle/high income). Educational levels were dichotomized as: high school, graduate or less. Marital status included: never married, married/living with partner, and divorced/separated/widowed.

#### Calculation of the SII

SII Complete blood counts were performed using automated hematology analyzers (Beckman Coulter DxH 800) to determine lymphocyte, neutrophil, and platelet counts in units of 10^3^ cells/μl. In line with prior reports^7^, The SII was computed as platelet count × neutrophil count/lymphocyte count.

#### OAB and eGFR

OAB was characterized by an overactive micturition reflex manifesting as urge urinary incontinence (UUI) and nocturia based on questionnaire responses. Trained staff conducted in-person interviews using standardized questionnaires. UUI presence and severity were determined via two questions: “During the past 12 months, have you leaked or lost control of even a small amount of urine with an urge or pressure to urinate and you could not get to the toilet fast enough?”, “How frequently does this occur?” Nocturia burden was evaluated using the question: “During the past 30 days, how many times per night did you most typically get up to urinate, from the time you went to bed at night until the time you got up in the morning” Additionally, the Overactive Bladder Symptom Score (OABSS) was employed, with an overall score ≥ 3 indicating OAB^19^. This validated measure aligned with prior applications of NHANES data^20,21^. Glomerular filtration rate (eGFR) was estimated using the 2009 Chronic Kidney Disease Epidemiology Collaboration (CKD-EPI) equation^22^.

### Statistical analysis

All statistical analyses adhered to Centers for Disease Control and Prevention guidelines, employing appropriate NHANES weights to account for the complex, multistage clustered sampling design. Continuous variables are reported as weighted means with standard errors, and categorical variables as proportions. Differences between groups were assessed using weighted Student’s t-tests for continuous variables and weighted χ^2^ tests for categorical variables. Given skewed distributions according to normality testing, log-transformed SII (log_10_(SII)) values were utilized. SII was also categorized into quartiles, designating the lowest quartile as reference. Age-standardized prevalence estimates and 95% CIs were calculated for each category of SII level.

Survey-weighted multivariable logistic regression modeled independent associations between SII and overactive bladder risk upon adjusting for potential demographic confounding factors and estimated glomerular filtration rate. Stratified analyses by age, sex, race, marital status, educational levels, poverty ratio examined susceptibility of subpopulations to demographic-related disparities. Interaction significance was estimated via product term *P* values assessing SII modification by stratified factors.

Statistical tests were 2-sided, and statistical significance was set at *P* < 0.05. All analyses were performed with R software, version 4.2.1 and Free Statistics software, version 1.8.

### Ethics statement

The survey protocol for the NHANES was approved by CDC’s National Center for Health Statistics Institutional Research Ethics Review Board. All participants provided written informed consent, and the study was approved by the NCHS Research Ethics Review Board (https://wwwn.cdc.gov/nchs/nhanes/default.aspx). Human subjects were not involved in this study.

## Result

### Baseline characteristics of participants

Table 1 shows the results of comparing levels of the SII and key demographic variables between subjects with and without OAB. The table first indicates that among all participants (n = 31,881), average SII was 548.15 and significantly higher in the OAB group compared to non-OAB (594.26 vs. 538.98, *P* < 0.001). A similar significant difference was seen for average age, with OAB patients averaging 57.67 years versus 45.70 years for those without. Dietary quality as assessed by the Healthy Eating Index did not significantly differ between the two groups. Regarding other characteristics, there was a higher proportion of females in the OAB group (62.68% vs. 49.29% non-OAB, *P* < 0.001). Race and ethnicity breakdown revealed White and Black people comprised the majority. Marital status, educational levels, poverty ratio, and prevalence of comorbidities including hypertension, coronary heart disease, and diabetes mellitus all showed significant associations with OAB presence.

**Table 1:** Baseline characteristics of participants (n = 31,881).

	Total (n = 31,881)	OAB		*P* value
		No (n = 25,186)	Yes (n = 6695)	
Age	47.68 (0.25)	45.70 (0.24)	57.67 (0.37)	< 0.0001
Gender				< 0.0001
Male	15,675 (48.49)	12,976 (50.71)	2699 (37.32)	
Female	16,206 (51.51)	12,210 (49.29)	3996 (62.68)	
Race				< 0.0001
White	14,007 (68.21)	11,244 (68.84)	2763 (65.08)	
Black	6627 (10.71)	4774 (9.54)	1853 (16.56)	
Mexican	5036 (8.50)	4030 (8.68)	1006 (7.62)	
Other	6211 (12.58)	5138 (12.94)	1073 (10.74)	
Marital status				< 0.001
Never married	5669 (18.39)	4825 (19.60)	844 (12.34)	
Married/living with a partner	19,144 (62.71)	15,545 (63.64)	3599 (58.18)	
Divorced/separated/widowed	7054 (18.86)	4806 (16.76)	2248 (29.48)	
Educational levels				< 0.0001
< High school	3208 (4.97)	2163 (4.20)	1045 (8.88)	
High school	11,821 (33.84)	8983 (32.31)	2838 (41.64)	
> High school	16,829 (61.14)	14,023 (63.49)	2806 (49.48)	
Poverty ratio				< 0.0001
< 1	5963 (13.08)	4379 (12.79)	1584 (20.09)	
1–5	18,056 (55.59)	14,239 (58.74)	3817 (62.62)	
> 5	5301 (24.95)	4608 (28.47)	693 (17.30)	
Healthy eating index	50.96 (0.21)	50.96 (0.22)	50.98 (0.34)	0.96
Hypertension				< 0.0001
No	18,206 (61.52)	15,728 (65.47)	2478 (41.70)	
Yes	13,672 (38.47)	9456 (34.53)	4216 (58.30)	
Coronary heart disease				< 0.001
No	30,430 (96.25)	24,312 (97.26)	6118 (92.80)	
Yes	1330 (3.47)	813 (2.74)	517 (7.20)	
Diabetes				< 0.0001
No	22,561 (76.74)	18,883 (80.51)	3678 (62.93)	
IFG	1491 (4.53)	1157 (4.40)	334 (5.49)	
IGT	1214 (3.37)	931 (3.31)	283 (3.90)	
Diabetes	6047 (14.22)	3853 (11.77)	2194 (27.68)	
SII	548.15 (3.46)	538.98 (3.45)	594.26 (7.77)	< 0.0001

*OAB* overactive bladder, *IFG* impaired fasting glucose, *IGT* impaired glucose tolerance, *SII* systemic immune-inflammation index.

*Data were presented as weighted percentages or means (95% confidence intervals).

### Association between SII and OAB

Table 2 presents results from three logistic regression models assessing the association between log_10_-transformed SII (log_10_(SII)) and OAB risk. The crude model showed 26% increased odds of OAB per unit increase in log_10_(SII) (odds ratio (OR) 1.26, 95% confidence interval (CI) 1.15, 1.37). Model 1 adjusted for sociodemographic factors—age, sex, race, educational levels, poverty ratio and marital status—and demonstrated a 25% higher OAB risk with rising log_10_(SII) (OR 1.25, 95% CI 1.15–1.37). Model 2 further controlled for kidney function and diabetes status, yet a two-fold log_10_(SII) increment retained a significantly elevated 18% odds of OAB (OR 1.18, 95% CI 1.08–1.28).

**Table 2:** Association between SII and OAB.

	Crude model	Model 1	Model 2
	OR (95%CI)	OR (95%CI)	OR (95%CI)
Log_10_(SII)	1.26 (1.15, 1.37)	1.25 (1.15, 1.37)	1.18 (1.08, 1.28)
SII quartiles			
Q1	Reference	Reference	Reference
Q2	0.92 (0.82, 1.03)	1.00 (0.88, 1.14)	0.99 (0.87, 1.13)
Q3	0.98 (0.88, 1.11)	1.06 (0.93, 1.20)	1.03 (0.90, 1.17)
Q4	1.36 (1.20, 1.54)	1.39 (1.21, 1.59)	1.28 (1.12, 1.47)
*P* for trend	< 0.001	< 0.001	< 0.001

Crude Model did not adjust for any confounding factors.

Model 1: Adjust for gender, age, race, educational levels, poverty ratio, marital status.

Model 2: Adjust for gender, age, race, educational levels, poverty ratio, marital status, kidney function, diabetes status.

*OR* odds ratio, *CI* confidence interval, *Log*_*10*_*(SII)* log_10_-transformed systemic immune-inflammation index.

SII was also analyzed categorically by quartiles, with the first quartile as reference. Compared to the lowest quartile, the highest SII quartile had a 36% increased OAB risk in the crude model (OR 1.36, 95% CI 1.20–1.54), which persisted at 39% higher odds after adjusting for sociodemographics in Model 1 (OR 1.39, 95% CI 1.21–1.59), and 28% elevated risk in the fully adjusted Model 2 (OR 1.28, 95% CI 1.12–1.47). A significant dose–response trend was observed across increasing SII quartiles in all three models (*P* for trend < 0.0001).

**Figure 2:**
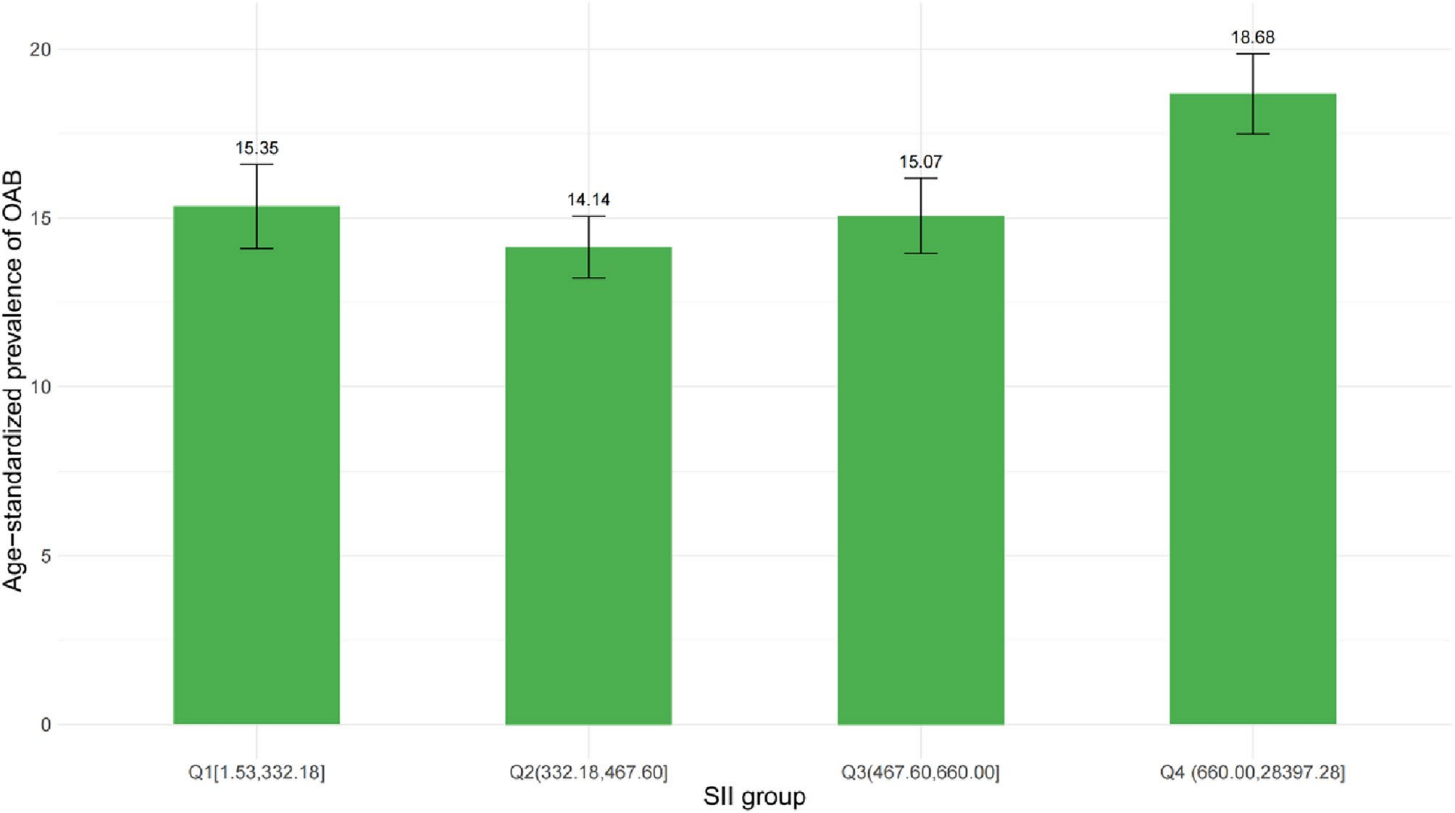
Age-adjusted prevalence of OAB in different levels of SII. Numbers at the top of the bars represent the weighted percentage. Bar whiskers represent the 95% confidence level.

Figure 2 presents the age-standardized prevalence of OAB across quartiles of the SII. A clear positive trend is observed, with the prevalence of OAB increasing monotonically from the lowest SII quartile (Q1: 15.35%) to the highest quartile (Q4: 18.68%). The second-lowest SII quartile (Q2) exhibits an age-standardized OAB prevalence of 14.14%, while the third quartile (Q3) demonstrates a prevalence of 15.07%, incrementally higher than Q2 but lower than the highest quartile Q4.

### Subgroup analysis

**Figure 3:**
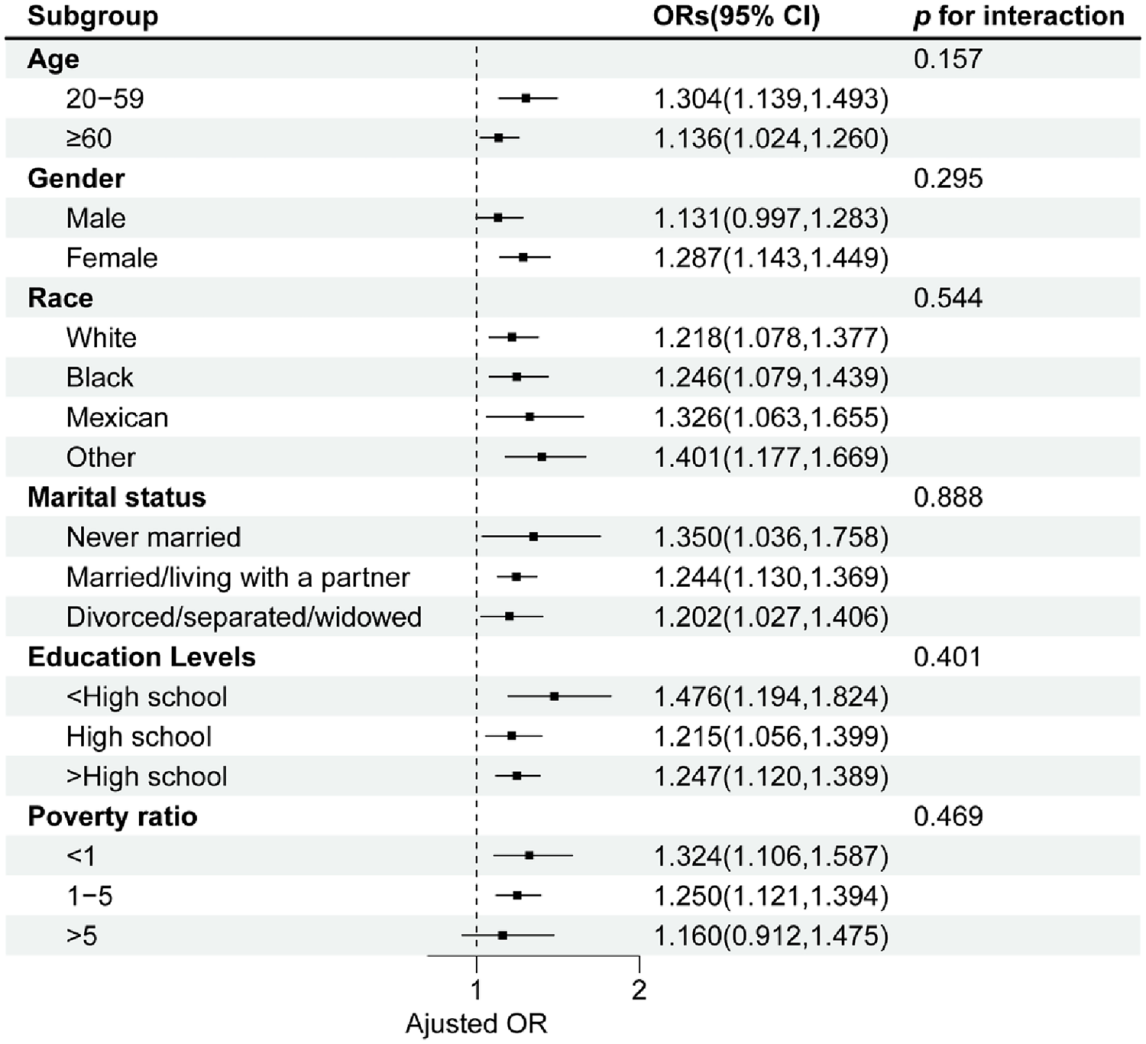
Subgroup analysis of the association of SII and OAB. ORs were calculated for each tenfold increase in SII. Each stratification was adjusted for gender, age, race, educational levels, poverty ratio, marital status, kidney function, diabetes status.

Figure 3 reports the results of the weighted logistic regression analysis of the relationship between SII and OAB risk for each stratification factor. For age group, individuals aged 20–59 had 30.4% higher odds of OAB compared to the reference (OR 1.304, 95% CI 1.139–1.493). Those ≥ 60 showed 13.6% increased risk (OR 1.136, 95% CI 1.024–1.260). By gender, females had 28.7% greater odds versus the reference (OR 1.287, 95% CI 1.143–1.449). Regarding race, Mexican participants exhibited 32.6% higher risk (OR 1.326, 95% CI 1.063–1.655). Black people (OR 1.246, 95% CI 1.079–1.439) and others (OR 1.401, 95% CI 1.177–1.669) also demonstrated elevated odds. Marital status stratified analyses showed 35.0% higher odds for those never married (OR 1.350, 95% CI 1.036–1.758), and 24.4% increased risk for married individuals (OR 1.244, 95% CI 1.130–1.369). The results of the subgroup analyses of educational levels and poverty ratio remain robust and stable, with the highest risk observed for those with less than a high school education (OR 1.476, 95% CI 1.194–1.824) and poverty ratio < 1 (OR 1.324, 95% CI 1.106–1.587), respectively. No interacting effects were detected (all *P* for interactions > 0.05, Fig. 3).

## Discussion

In our study sample, which exhibits national representativeness of the US adult population, we observed a positive correlation between SII levels and OAB. The age-standardized prevalence of OAB increased monotonically across quartiles of SII, rising from 15.35% in the lowest quartile to 18.68% in the highest quartile (Fig. 2). Findings from subgroup analyses and interaction testing further suggest that this positive association between elevated SII and increased OAB risk holds stable across various demographics, including age, sex, race, marital status, educational levels, and poverty ratio. These results indicate that the systemic inflammatory state, as reflected by higher SII, may be an important contributing factor to the development and progression of overactive bladder. The SII, as an objective and easily accessible biomarker, could provide valuable insights into the underlying pathophysiology linking inflammation and bladder dysfunction.

Multiple studies have reported a correlation between inflammation and the occurrence and progression of OAB. Prolonged inflammation can induce alterations in bladder function and heightened sensitivity, leading to the manifestation of OAB symptoms^12,23,24^, and molecular imbalances within inflammatory proteins may contribute to the development of OAB^25^. Tyagi et al.^26^ found that monocyte chemotactic protein-1 (MCP-1), soluble fraction of the CD40 ligand (sCD40L), growth-related oncogene GRO-α in the urine of OAB patients, macrophage inflammatory protein (MIP-1β), IL-5, IL-12p70/p40, epidermal growth factor (EGF), and other cytokine levels were elevated, suggesting the presence of infiltration of neutrophils, eosinophils, and mast cells in the bladder of OAB patients. Furthermore, studies have revealed an increase in the number and activity of mast cells, T-lymphocytes, and B-lymphocytes within the detrusor muscle of OAB patients^27–29^. The release of these inflammatory cells and mediators leads to an inflammatory response in the bladder tissue, which in turn triggers the symptoms of OAB. All of these studies suggested that inflammation is associated with OAB. And SII, as a systemic indicator of immune inflammation, can reflect the level of inflammation in the organism. Therefore, SII may be a good tool to help distinguish OAB and evaluate treatment effectiveness.

SII is calculated by multiplying the platelet count by the neutrophil count and dividing by the lymphocyte count. In a recent study, Kim et al.^30^ observed that women with OAB exhibited significantly higher neutrophil-to-lymphocyte ratios (NLR) compared to those without OAB. Furthermore, they identified a strong correlation between NLR and the severity of OAB symptoms, as measured by the OABSS. Moreover, considering the calculation formula of the SII, it becomes evident that the NLR has a strong correlation with the SII score. This suggests that SII may also be related to the severity of OAB, and our findings confirm this. Increasingly, platelets have been found to be clinically important for the diagnosis and treatment of inflammation. Platelets contain a large number of molecules associated with immune-inflammatory response such as PF4, CD40L, PDGF, TGF-β, and MIP-1α, which interacts with inflammatory cells and thus promotes inflammation^31,32^. Thus compared to NLR, SII might be able to represent the systemic immune-inflammatory response more comprehensively. This suggests that the SII may also be associated with the severity of OAB. Another study by Lin et al.^33^ demonstrated that low-intensity extracorporeal shock wave therapy (LI-ESWT) can mitigate inflammatory responses, stimulate angiogenesis, promote cell proliferation and differentiation, reduce urinary leakage, and improve OAB symptoms. Moreover, Chen et al.^34^ found that antibiotic therapy in women with refractory detrusor overactivity (DO), which is a subtype of OAB, led to a reduction in cytokines associated with innate immune system activation. Their study demonstrated that attenuating the immune-inflammatory response was also associated with symptom improvement in these individuals. This suggests that immune-inflammatory response may play a potential role in the pathogenesis of refractory DO^34^. Overall, these studies provide valuable insights into the relationship between inflammation and OAB. In our study, SII, which reflects systemic inflammation status, may serve as a bridge connecting inflammation responses to OAB symptoms and severity.

This study represents the first investigation into the correlation between SII and OAB, suggesting the potential of SII as a tool for OAB diagnosis and assessment. However, certain limitations should be acknowledged. Firstly, the cross-sectional design of the study precludes determining a causal relationship between SII and OAB. Secondly, the susceptibility of neutrophil, lymphocyte, and platelet counts to variability may result in selection bias. Thirdly, although confounding factors were controlled for, the influence of potential residual confounders cannot be fully ruled out. Additionally, the lack of laboratory tests in the NHANES dataset limited OAB diagnosis, which solely relied on the Overactive Bladder Symptom Score (OABSS). Lastly, symptoms collection through questionnaires may be susceptible to recall bias. Prospective studies with larger sample sizes are warranted to clarify the causal associations between these variables and evaluate SII's value in OAB management longitudinally.

## Conclusion

Our findings imply that increased SII levels are linked to OAB. This suggests that higher levels of SII may contribute to an increased incidence of OAB and could potentially be used as a cost-effective and direct biomarker for the detection of OAB. To confirm our findings, more large-scale prospective investigations are needed.

## Data Availability

The original contributions presented in the study are included in the article/Supplementary Material. Further inquiries can be directed to the corresponding authors.
